# Infrastructure of pharmacies of the primary health care in the Brazilian Unified Health System: Analysis of PNAUM – Services data

**DOI:** 10.11606/S1518-8787.2017051007120

**Published:** 2017-09-22

**Authors:** Silvana Nair Leite, Fernanda Manzini, Juliana Álvares, Augusto Afonso Guerra, Ediná Alves Costa, Francisco de Assis Acurcio, Ione Aquemi Guibu, Karen Sarmento Costa, Margô Gomes de Oliveira Karnikowski, Orlando Mário Soeiro, Mareni Rocha Farias

**Affiliations:** I Departamento de Ciências Farmacêuticas. Universidade Federal de Santa Catarina. Florianópolis, SC, Brasil; II Programa de Pós-Graduação em Farmácia. Universidade Federal de Santa Catarina. Florianópolis, SC, Brasil; III Departamento de Farmácia Social. Faculdade de Farmácia. Universidade Federal de Minas Gerais. Belo Horizonte, MG, Brasil; IV Instituto de Saúde Coletiva. Universidade Federal da Bahia. Salvador, BA, Brasil; V Departamento de Saúde Coletiva. Faculdade de Ciências Médicas. Santa Casa de São Paulo. São Paulo, SP, Brasil; VI Núcleo de Estudos de Políticas Públicas. Universidade Estadual de Campinas. Campinas, SP, Brasil; VII Programa de Pós-Graduação em Saúde Coletiva. Departamento de Saúde Coletiva. Faculdade de Ciências Médicas. Universidade Estadual de Campinas. Campinas, SP, Brasil; VIII Programa de Pós-Graduação em Epidemiologia. Faculdade de Medicina. Universidade Federal do Rio Grande do Sul. Porto Alegre, RS, Brasil; IX Faculdade de Ceilândia. Universidade de Brasília. Brasília, DF, Brasil; X Faculdade de Ciências Farmacêuticas. Pontifícia Universidade Católica de Campinas. Campinas, SP, Brasil

**Keywords:** Pharmaceutical Services, supply & distribution, Infrastructure, Primary Health Care, Health Services Research, Unified Health System, Assistência Farmacêutica, provisão & distribuição, Infraestrutura, Atenção Primária à Saúde, Pesquisa sobre Serviços de Saúde, Sistema Único de Saúde

## Abstract

**OBJECTIVE:**

To characterize the infrastructure of the primary health care pharmacies of the Brazilian Unified Health System, aiming at humanizing the offered services.

**METHODS:**

This is a cross-sectional study, of quantitative approach, from data obtained in the *Pesquisa Nacional de Acesso, Utilização e Promoção do Uso Racional de Medicamentos – Serviços, 2015* (PNAUM – National Survey on Access, Use and Promotion of Rational Use of Medicines – Services, 2015). Information on 1,175 pharmacies/dispensing units were gathered from direct observation and assessment of dispensing units installations conducted by trained researchers who used a standardized form. The analyzed variables refer to the physical structure of pharmacies or medicine dispensing units of the health units under research.

**RESULTS:**

The pharmacy area was greater than 14 m^2^ in 40.3% of the sampled units, highlighting those from Midwest (56.9%) and Southeast (56.2%) regions and those of Northeast, with only 23.3%. About 80.2% units had waiting rooms with chairs for patients, 31.8% of them had dispensing areas inferior to 5m^2^, while in 46.2% these areas were superior to 10m^2^. Bars were found in service counters in 23.8% of health units, thus separating the patient from the professional; 44.1% had internet access. In most units, the area of medicine storage had no refrigerator or freezer for their exclusive storage and 13.7% had a specific room for pharmaceutical consultation.

**CONCLUSION:**

Aiming at achieving care humanization and improving working conditions for professionals, the structuring of the environment of pharmacy services is necessary. This would contribute to the better qualification of pharmacy services, comprising more than medicine delivery. Data on the Northeast region indicated less favorable conditions to the development of adequate dispensing services. Based on the panorama pointed out, we suggest the expansion of stimulus concerning the physical structure of pharmaceutical services, considering regional specificities.

## RESUMO

**OBJETIVO:**

Caracterizar a infraestrutura das farmácias da atenção básica do Sistema Único de Saúde, visando condição para a humanização dos serviços ofertados.

**METODOS:**

Trata-se de estudo transversal, de abordagem quantitativa, a partir de dados obtidos da Pesquisa Nacional sobre Acesso, Utilização e Promoção do Uso Racional de Medicamentos – Serviços, 2015. As informações de 1.175 farmácias/unidades de dispensação foram oriundas de observação direta e mensuração das instalações das unidades de dispensação realizadas por pesquisadores treinados e uso de formulário padronizado. As variáveis analisadas referem-se à estrutura física das farmácias ou unidades de dispensação de medicamentos das unidades de saúde amostradas.

**RESULTADOS:**

A área da farmácia foi superior a 14 m^2^ em 40,3% das unidades pesquisadas, com destaque para as regiões Centro-Oeste (56,9%) e Sudeste (56,2%) e Nordeste com apenas 26,3%. A sala de espera possuía cadeiras para os usuários (80,2%), em 31,8% a área de dispensação era inferior à 5m^2^ e em 46,2% foi superior a 10m^2^. Foram encontradas grades no guichê de atendimento, separando o atendente do usuário em 23,8% das unidades; havia acesso à internet em 44,1%. Na maioria das unidades, a área destinada ao armazenamento de medicamentos não possuía refrigerador ou geladeira para o seu armazenamento exclusivo e 13,7% apresentavam área para consulta farmacêutica.

**CONCLUSÕES:**

É necessária a estruturação da ambiência dos serviços de farmácia visando a humanização do atendimento e a melhoria das condições de trabalho aos profissionais. Isso propicia melhor qualificação do serviço de farmácia para além da entrega do medicamento. Os dados apresentados pela região Nordeste indicam condições menos favoráveis ao desenvolvimento de serviços de dispensação adequados. Com base no panorama apontado, sugere-se a ampliação dos incentivos para a estruturação física dos serviços farmacêuticos, levando em consideração as especificidades regionais.

## INTRODUCTION

The Brazilian Ministry of Health adopted the *Estratégia Saúde da Família* (ESF – Family Health Strategy) as a model for the reorganization of primary health care in the Country, by following the principles of the Brazilian Unified Health System (SUS). ESF is structured to be the preferred contact of patients, the main entry to SUS, and the communication center with the health care network, based on the relationship between the patient and the multidisciplinary team. The structure of the *Unidades Básicas de Saúde* (UBS – Basic Health Units), destined to the services of Family Health teams, might contribute to changes in the health practices of these professionals, promoting resolutive and humanized services, and ensuring care continuity^13^. Medicine dispensing is one of the services conducted in UBS.

Data from the *Cadastro Nacional de Estabelecimentos de Saúde* (CNES – National Registration of Health Establishments) indicate a number of 51,184 health units and 10,054 community health centers in Brazil, with an increase of 13% in the number of health units and of 75% in pharmacists registered in these establishments between 2008 and 2013^7^. The Brazilian Ministry of Health recommends the existence of a pharmacy in each UBS, but the service is not mandatory in cities where medicine dispensing is carried out in a centralized way^13^.

Pharmacies – located in health units or where it is not necessary to share spaces and structures with other health services –, must have a physical infrastructure, human and material resources that allow the integration of the services and the development of full and efficient actions on pharmaceutical services. This, they ensure the quality of medicines, humanized care, optimization of resources, and effective implementation of actions that improve health care conditions^14^.

In 2006, Araújo & Freitas^3^ pointed out that the pharmacies of UBS generally occupied an area of around 20 m^2^, structured as storage area, and professionals carried out dispensation through an opening in the partition between the pharmacy environment and the one destined to the patient. The authors state that these characteristics did not change regardless of the UBS structure (old, new, or reformed). An evaluation conducted by the Pan American Health Organization and the Brazilian Ministry of Health^21^ identified problems regarding medicine stocking in areas for storage and medicine dispensing of health units and pharmaceutical supply centers from all regions of the Country.

Alencar & Nascimento^1^ indicate that most UBS do not have a specific area for pharmacy, only cupboards or rooms for medicine storage. According to these authors, these spaces do not meet the criteria of good storage practices, considering they observed excessive brightness, little sanitation, and even the presence of insects.

Documents of the Brazilian Ministry of Health guide conception and structuring of pharmacies of SUS, such as the Manual of Physical Structure of Health Units (1st edition – 2006^12^ and 2nd edition – 2008^13^), the Ordinance GM/MS no. 1.903/2013, and the Guidelines for Pharmacy Structuring in the Brazilian Unified Health System^14^. The needed structure is defined according to the services to be performed, as seen in two pharmacy models: Model A (area for medicine dispensing, dose fractioning, and stocking room) and Model B (includes a room for pharmacotherapeutic care), and also by the number of Family Health teams of the UBS.

Even after the publication of several norms regarding the structuring of pharmacies of SUS, the profile pointed by Araújo & Freitas^3^ is still found in several UBS, possibly because of the predominance of technical and operational activities in pharmaceutical services. Strengthening the understanding of medicine as a product – by a schematic model called operating cycle of pharmaceutical services – reinforced that medicines (as a technology) are more privileged than individuals in certain conceptions of pharmaceutical services ^10^.

Reinforcing the importance of the structuring of pharmaceutical services, in 2008, the Brazilian Ministry of Health, the *Conselho Nacional de Secretários de Saúde* (CONASS – National Council of Secretaries of Health), and the *Conselho Nacional de Secretarias Municipais de Saúde* (CONASEMS – National Council of Municipal Secretaries of Health) signed the *Nota Técnica Conjunta* (NT – Joint Technical Note)^a^, which deals with the qualification of pharmaceutical services. According to NT, in the context of rational and safe use of medicines, access cannot be restricted to them as it must comprise actions in pharmaceutical services and of an entire set of health care initiatives from qualified services, demanding a differentiated pharmacy structure.

Considering the physical space as a social, professional, and interpersonal space, the environment of these services follows some principles: ensuring the privacy and individuality of the subjects involved; allowing the production of subjectivities from actions and reflections on the working processes; being used as a facilitator of the working process, favoring the optimization of resources, and an humanized, resolutive, and welcoming service^15^. To do so, the physical space of services must be redefined to offer humanized care^16^, thus improving the services of several levels of health care^4^ ^,^ ^22^ ^,^ ^24^ and the process of user care^28^.

The physical structure of a service directly affects the working conditions of professionals and influences the health and developed health practices. Maciel, Santos, and Rodrigues^11^ point out that problems in the organization of work and in the physical conditions of the UBS lead workers to a forced adaptation, compromising the quality of care. They also point out the excess of demand as one of the main factors that impair the quality of the provided care.

Since 2010, the financial resources available for pharmaceutical services – previously destined to medicine acquisition –, could also support structuring actions in pharmacies of SUS and the qualification of pharmaceutical services of Basic Pharmaceutical Services (until 15% of the sum of state and municipal funds)^17^. Initiatives, such as the *Programa Nacional de Qualificação da Assistência Farmacêutica no Âmbito do SUS* (QUALIFAR-SUS – National Program for Qualification of Pharmaceutical Services of SUS) and the *Programa de Requalificação de Unidades Básicas de Saúde* (Requalifica UBS – Brazilian Program of Requalification of Primary Health Care Units), have enabled the provision of resources for the structuring of the services in prioritized cities.

The *Pesquisa Nacional sobre Acesso, Utilização e Promoção do Uso Racional de Medicamentos – Serviços* (PNAUM – National Survey on Access, Use and Promotion of Rational Use of Medicines – Services) aimed to characterize the organization of pharmaceutical services in the primary health care of SUS – for promoting the access and rational use of medicines – as well as to identify and discuss the factors that interfere in the consolidation of pharmaceutical services in the cities.

This study is part of PNAUM – Services and aimed to characterize the physical structure of pharmacies of primary health care of SUS, for humanizing the offered services and working conditions of health professionals of these places.

## METHODS

PNAUM is a cross-sectional study of exploratory and evaluative nature, consisting of an information survey in a representative sample of primary health care services in cities of different sizes from the five Brazilian regions. Several study populations were considered in the sampling plan, with samples stratified according to the regions, which constitute the study domains. For this study, we used information gathered from direct observation and assessment of dispensing units’ installations, both conducted by trained researchers with a standardized form. The analyzed variables refer to the physical structure of pharmacies or medicine dispensing units of the sampled health units. The sampling was estimated in 300 cities (120 in each region, including capitals, larger cities, and smaller ones) and in 1,541 health units (considering the sampling of units by city).

Data were collected from July to December 2014. The methodology of PNAUM – Services, as well as the sampling process and the conduction of the field work, are described in detail by Álvares et al.^2^ The results are presented according to five Brazilian geographic regions.

Data analysis was performed using the SPSS® software, version 22, from a complex sample plan. Descriptive analyses were presented in percentages. Statistically significant differences were considered when p<0.01 for Pearson’s Chi-square test. This research was approved by the National Research Ethics Committee with *Certificado de Apresentação para Apreciação Ética* (CAAE – Certificate of Presentation for Ethical Consideration) no. 18947013.6.0000.0008 and all participants signed the informed consent form.

## RESULTS

We analyzed around 1,175 pharmacies/medicine dispensing units (86.3% of the estimated sample) of the primary health care network from all regions of the Country, totaling 273 cities (91% of the estimated sample).

The average area of pharmacies was greater than 14 m^2^ in 40.3% of the surveyed units, especially in the Midwest and Southeast regions, with 56.9% and 56.2%, respectively (Table 1).

**Table 1:** Characterization of the infrastructure of pharmacies and dispensing units, according to Brazilian regions. National Survey on Access, Use and Promotion of Rational Use of Medicines – Services, 2015.

Variable	Brazilian regions % (95%CI)					Total

	North	Northeast	Midwest	Southeast	South	
Pharmacy total area*						
≤ 14 m^2^	68.0 (61.3–74.0)	73.7 (63.8–81.6)	43.1 (31.4–55.8)	43.8 (32.1–56.3)	60.2 (48.2–71.1)	59.7 (53.4–65.7)
> 14 m^2^	32.0 (26.0–38.07)	26.3 (18.4–36.2)	56.9 (44.2–68.6)	56.2 (43.7–67.9)	39.8 (28.9–51.8)	40.3 (34.3–46.6)
Waiting room for patients						
Yes, exclusive	13.1 (9.3–18.2)	26.0 (19.0–34.5)	48.6 (35.4–61.9)	53.4 (40.6–65.8)	28.4 (18.3–41.2)	35.2 (29.3–41.6)
Yes, shared	63.0 (56.5–69.1)	62.3 (53.9–70.0)	39.4 (28.3–51.6)	40.0 (29.1–51.9)	62.1 (50.1–72.7)	54.1 (48.3–59.7)
No	23.9 (18.7–30.0)	11.7 (7.8–17.2)	12.1 (6.5–21.4)	6.6 (3.4–12.3)	9.5 (4.6–18.7)	10.8 (8.4–13.7)
Area for medicine dispensing*						
Yes, exclusive	60.1 (53.5–66.4)	57.9 (49.6–65.8)	78.1 (64.2–87.6)	72.3 (49.6–65.8)	57.9 (49.6–65.8)	66.2 (61.3–70.7)
Yes, shared	39.3 (33.1–45.9)	39.8 (32.0–48.1)	21.8 (12.3–35.7)	27.6 (19.5–37.5)	26.8 (20.0–35.0)	32.7 (28.2–37.6)
No	0.6 (0.1–2.4)	2.3 (1.0–5.5)	0.1 (0.0–0.9)	0	0.9 (0.2–3.4)	1.1 (0.5–2.3)
Dispensing area*						
≤ 5 m^2^	24.9 (19.5–31.2)	44.5 (36.3–53.1)	18.0 (11.3–27.4)	28.7 (20.3–38.9)	18.8 (13.4–25.8)	31.8 (27.5–36.6)
> 5 m^2^	75.1 (68.8–80.5)	55.5 (46.9–63.7)	82.0 (72.6–88.7)	71.3 (61.1–79.7)	81.2 (74.2–86.6)	68.2 (63.4–72.5)
Dispensing area*						
≤ 10 m^2^	60.3 (53.5–66.7)	68.5 (58.5–77.0)	42.7 (30.3–56.1)	45.3 (33.1–58.0)	53.2 (42.1–64.0)	53.8 (48.4–59.1)
> 10 m^2^	39.7 (33.3–46.5)	31.5 (23.0–41.5)	57.3 (43.9–69.7)	54.7 (42.0–66.9)	46.8 (36.0–57.9)	46.2 (40.9–51.6)
Area for pharmaceutical consultation or pharmacotherapeutic follow-up*						
Yes, exclusive	2.1 (1.0–4.2)	3.0 (1.4–6.3)	13.6 (5.4–30.1)	18.6 (8.2–36.7)	5.1 (2.2–11.2)	8.8 (4.9–15.1)
Yes, shared	4.8 (2.6–8.8)	2.4 (0.5–10.3)	3.9 (1.8–8.2)	4.4 (1.7–10.8)	11.6 (3.7–31.2)	4.9 (2.6–8.8)
No	93.1 (89.0–95.8)	94.6 (88.5–97.5)	82.5 (68.1–91.2)	77.0 (60.3–88.1)	83.3 (66.8–92.5)	86.4 (79.9–90.9)
Area for medicine storage apart from the dispensing area	22.3 (17.4–28.1)	18.4 (11.8–27.7)	44.2 (31.5–57.6)	56.2 (43.8–67.9)	50.3 (39.9–60.6)	37.3 (31.4–43.5)
Area for medicine storage*						
Exclusive	73.4 (67.3–78.7)	77.3 (70.3–83.0)	91.4 (84.6–95.4)	88.6 (81.4–93.2)	81.3 (74.2–86.7)	82.0 (78.4–85.1)
Shared	26.6 (21.3–32.7)	22.7 (17.0–29.7)	8.6 (4.6–15.4)	11.4 (6.8–18.6)	18.7 (13.3–25.8)	18.0 (14.9–21.6)
Storage area*						
≤ 10 m^2^	62.3 (55.5–68.6)	73.9 (64.4–81.6)	41.9 (32.0–52.5)	42.5 (31.0–54.8)	57.9 (46.1–68.9)	58.1 (52.0–64.0)
> 10 m^2^	37.7 (31.4–44.5)	26.1 (18.4–35.6)	58.1 (47.5–68.0)	57.5 (45.2–69.0)	26.1 (18.4–35.6)	41.9 (36.0–48.0)
Area for storage of expired/unsuitable for use products*	32.4 (26.5–38.9)	15.0 (9.8–22.3)	54.8 (41.6–67.4)	63.2 (50.8–74.1)	45.9 (35.3–56.8)	38.9 (33.4–44.6)
Has electric generator	4.1 (2.1–7.7)	1.0 (0.3–3.9)	5.8 (2.2–14.1)	3.7 (1.7–8.0)	2.9 (0.8–9.8)	2.7 (1.7–4.3)

Source: PNAUM – Services, 2015.

* p > 0.01

We found waiting rooms destined to patients in 89.3% health units under research, and around 54.1% of them were exclusive to this activity (Table 1).

Most units has an area destined to medicine dispensing (98.9%), and 66.2% of them were exclusive to this activity. In 31.8% of units, this area was inferior to 5 m^2^, and 46.2% of them had more than 10 m^2^. In the Northeast region, 44.5% of the units had dispensing area inferior to 5 m^2^, while units from Midwest and Southeast regions had 57.3% and 54.7% dispensing areas with more than 10 m^2^, respectively (Table 1). Only 37.3% of the units had an area destined to medicine storage apart from the medicine dispensing area. In most units (58.1%), storage area was inferior to 10m^2^ and 82.0% of them used it exclusively for medicine storage (Table 1).

We found areas destined to pharmaceutical consultation or pharmacotherapeutic follow-up only in 13.7% of the units, and the Southeast region had them in 23% of its units (Table 1).

In 38.9% of units, we observed an area destined to the storage of expired or unsuitable for use products, a percentage of only 15.0% in the North region. Most units did not have electric generator (Table 1).

The waiting room of most units had chairs (80.2%), a billboard (61.7%), educational materials on health (58.5%), drinking fountain/water purifier/filter (68.2%), access to restrooms (81.0%), and protection from the sun and rain (96.6%). Only 30% of them had a television (Table 2).

**Table 2:** Characterization of the waiting area of pharmacies and dispensing units, according to Brazilian regions. National Survey on Access, Use and Promotion of Rational Use of Medicines – Services, 2015.

Variable	Brazilian regions % (95%CI)					Total

	North	Northeast	Midwest	Southeast	South	
Has password system for meeting patients*	2.1 (0.7–6.2)	1.3 (0.5–3.7)	0.6 (0.1–2.8)	21.3 (8.3–44.7)	14.1 (9.6–20.1)	10.0 (5.0–19.0)
Has chairs*	80.9 (74.1–86.2)	68.8 (59.8–76.5)	85.5 (72.1–93.1)	88.4 (80.2–93.4)	88.1 (81.4–92.6)	80.2 (75.7–84.1)
Has a billboard*	57.3 (49.7–64.6)	45.7 (36.8–54.8)	78.9 (69.8–85.7)	75.4 (64.8–83.6)	67.3 (56.8–76.3)	61.7 (56.2–66.9)
Has a television*	28.9 (22.5–36.3)	18.7 (13.2–25.8)	30.5 (19.3–44.5)	37.6 (24.9–52.2)	41.1 (31.5–51.4)	30.0 (24.8–35.8)
Provides educational materials on health	61.2 (53.8–68.1)	45.5 (36.7–54.5)	68.2 (55.5–78.6)	66.7 (51.1–79.3)	67.6 (54.3–78.6)	58.5 (52.4–64.4)
Has a water fountain/water purifier/filter	70.2 (62.8–76.6)	67.9 (59.4–75.4)	70.6 (49.0–85.8)	70.4 (57.4–80.7)	63.3 (50.8–74.3)	68.2 (62.5–73.5)
Has access to restrooms	84.8 (78.6–89.4)	89.7 (82.8–91.4)	66.1 (43.3–83.3)	73.6 (56.2–85.9)	79.3 (69.2–86.6)	81.0 (74.3–86.3)
Has protection from the sun and rain	97.5 (93.6–99.0)	94.6 (85.8–98.1)	98.5 (93.9–99.6)	97.9 (93.0–99.4)	97.4 (92.1–99.1)	96.6 (93.3–98.3)

Source: PNAUM – Services, 2015.

* p > 0.01

We found that 23.7% of the units had individual service counters with chairs to sit and that 59.0% had service counters without chairs. In 23.8% of units, we found service counter with bars that separated professionals from patients. The Midwest and Southeast regions had bars in 41.4% and 41.8% of the units, respectively. However, in the South, only 5.4% of units presented bars (Table 3).

**Table 3:** Characterization of the dispensing area of pharmacies and dispensing units, according to Brazilian regions. National Survey on Access, Use and Promotion of Rational Use of Medicines – Services, 2015.

Variable	Brazilian regions % (95%CI)					Total

	North	Northeast	Midwest	Southeast	South	
Has password system for meeting patients*	2.1 (0.9–5.1)	1.1 (0.3–3.4)	0.5 (0.1–2.4)	20.3 (8.0–42.7)	13.1 (9.0–18.6)	9.2 (4.6–17.4)
Has individual service counters/tables with chairs*	23.4 (18.2–29.4)	12.2 (7.1–20.3)	23.6 (15.0–35.2)	38.4 (24.5–54.6)	22.1 (12.6–35.7)	23.7 (17.8–30.8)
Has service counters without chairs*	48.6 (42.0–55.2)	38.7 (30.8–47.1)	68.0 (54.4–79.1)	76.7 (67.1–84.1)	73.8 (65.6–80.6)	59.0 (54.0–3.9)
Has service counters with bars that separate professionals from patients*	24.0 (19.2–29.5)	14.3 (8.9–22.1)	41.4 (26.3–58.3)	41.8 (30.3–54.2)	5.4 (3.1–9.0)	23.8 (19.8–28.2)
Has computerized system to register activities of pharmaceutical services*	11.4 (8.0–16.0)	15.3 (9.1–25.4)	57.8 (43.4–71.0)	57.7 (44.0–70.3)	66.6 (57.5–74.5)	39.4 (34.0–45.0)
Has a computer*	23.3 (18.8–28.4)	16.4 (10.0–25.7)	65.6 (51.8–77.1)	74.7 (64.9–82.5)	69.9 (61.3–77.2)	47.1 (41.8–52.5)
Has internet access*	19.3 (15.3–24.2)	16.4 (10.0–25.8)	69.6 (57.1–79.8)	66.7 (52.6–78.4)	66.9 (58.1–74.7)	44.1 (38.7–49.5)
Has a printer*	12.0 (8.5–16.7)	11.1 (5.5–21.0)	49.2 (36.8–61.6)	39.5 (27.0–53.5)	46.6 (36.2–57.2)	28.3 (23.0–34.2)
Has a phone*	4.7 (3.0–7.2)	8.2 (3.9–16.7)	50.5 (38.0–63.0)	55.4 (42.6–67.4)	48.3 (37.9–58.9)	31.9 (26.3–38.2)
Has a table for the professional*	72.6 (66.2–78.2)	63.3 (55.3–70.6)	94.3 (89.3–97.1)	91.0 (85.3–94.7)	81.1 (72.4–87.5)	77.6 (73.7–81.0)
Has a chair for the professional*	75.4 (69.2–80.7)	68.5 (60.8–75.3)	94.6 (89.7–97.3)	91.3 (84.4–95.3)	83.8 (75.4–89.7)	80.3 (76.5–83.7)
Has a chair for the patient	40.9 (34.6–47.6)	37.9 (30.0–46.5)	47.3 (34.8–60.2)	47.3 (33.9–61.1)	37.4 (27.4–48.7)	41.6 (35.7–47.7)
Controls the entry and transit of people*	44.4 (37.9–51.1)	64.7 (56.1–72.5)	54.0 (41.1–66.5)	54.2 (40.0–67.7)	79.1 (69.6–86.2)	61.5 (55.4–67.4)
Has air conditioning*	62.4 (55.9–68.6)	29.0 (21.2–38.1)	76.3 (67.1–83.5)	43.7 (31.8–56.3)	44.7 (34.1–55.7)	41.7 (36.5–47.2)

Source: PNAUM – Services, 2015.

* p > 0.01

The number of units that had computers in the medicine dispensing area was of 47.1%; around 44.1% of the units had access to the internet and 39.4% had a computerized system to register activities regarding pharmaceutical services. In the Northeast region, 16.4% of the units had access to the internet, similar to the situation found in the North (19.3%) (Table 3).

Most units had no phone, printer, and air conditioning in the medicine dispensing area; 9.2% of them had a password system for meeting patients (Table 3).

Although some of them had tables (77.6%) and chairs (80.3%) for professionals in the dispensing area, most units did not have chairs for patients (Table 3).

We often observed that the area for medicines storage had no exclusive refrigerator or freezer for medicine storage, and no digital thermometer to verify the temperature of the environment and the refrigerator. Most units controlled the entry and transit of people in this area (Table 4).

**Table 4:** Characterization of the storage area of pharmacies and dispensing units, according to Brazilian regions. National Survey on Access, Use and Promotion of Rational Use of Medicines – Services, 2015.

Variable	Brazilian regions % (95%CI)					Total

	North	Northeast	Midwest	Southeast	South	
Has refrigerator/freezer for the exclusive storage of medicines*	37.2 (31.0–43.8)	21.3 (16.1–27.6)	50.7 (36.6–64.7)	76.0 (65.9–83.9)	56.8 (46.7–66.4)	47.2 (42.0–52.5)
Has refrigerator/freezer for shared storage of medicines and other products and/or food	5.9 (3.4–10.0)	8.5 (5.3–13.4)	12.7 (7.6–20.6)	9.1 (5.5–14.7)	8.5 (5.3–13.4)	7.8 (5.9–10.1)
Controls the entry and transit of people	49.5 (42.9–56.1)	63.8 (55.3–71.5)	58.8 (46.0–70.6)	64.4 (49.5–76.9)	81.0 (73.8–86.6)	65.4 (59.4–70.7)
Has a digital thermometer to verify the temperature of the environment*	11.3 (7.9–16.0)	6.1 (3.2–11.4)	39.5 (25.0–56.0)	51.6 (38.3–64.7)	28.9 (19.2–41.0)	26.4 (21.5–32.0)
Has a digital thermometer to verify the temperature of the refrigerator*	25.0 (19.7–31.2)	16.2 (11.7–22.0)	43.5 (30.2–57.7)	71.4 (60.4–80.4)	50.2 (39.9–60.6)	41.3 (36.0–46.8)

Source: PNAUM – Services, 2015.

* p > 0.01

## DISCUSSION

Since the creation of the *Programa Saúde da Família* (PSF – Family Health Program), the number of health units has grown across the Country. Between 2008 and 2013, this rate was higher in the Northeast^7^. However, our results show that the infrastructure of pharmacies or medicine dispensing units are different between the regions, the most deficient infrastructure being those from the North and Northeast, especially regarding computerization and internet access.

Despite being an essential input to the solutions of health care, medicines were belatedly understood as part of the process of care and in the health system. The first National Drug Policy was published in 1998, while the *Política Nacional de Assistência Farmacêutica* (PNAF – National Policy of Pharmaceutical Services) was only published in 2004, presenting the concept of pharmaceutical services associated with medicine use in primary health care. This history allows us to understand the way that spaces and equipment regarding the pharmaceutical services of health units did not have adequate attention for a long period since these units were deployed, which is reflected until today.

We observed that around 60% of dispensing units had a minimum space of about 14 m^2^ for pharmacies; in the Northeast region, this space was inferior to 14 m^2^ in 80% of units. The space for medicine dispensing, which involves the patient-dispenser relationship, was inferior to 5 m^2^ in more than 40% of the units in the Northeast region, and many of these areas were shared with other activities, indicating inadequate conditions to develop dispensing services, as those advocated by Soares et al.^25^

Other concerning factor is the sanitation of pharmacies, as identified by Dias^9^: the author observed infiltration and molds on the walls, roof leaks, cracks in the floor, as well as the lack of brightness and ventilation.

The high percentage of units that did not have exclusive refrigerators for medicines, thermometers, and air conditioning is very alarming, considering Brazil has critical weather conditions for medicine conservation. Human NPH and regular insulin integrate the cast of medicines that should be available in SUS, according to Ordinance no. 2.583/2007. We wonder, thus, which is the real conservation situation of these medicines in units that have no refrigerator or that need to share its space with other products and/or food. A study^27^ has shown that inadequate storage of insulin at temperatures above 30°C weakens its potential and, consequently, its pharmacological action.

The lack of equipment regarding the provision of services, both for medicine care and patient care, is particularly concerning in the North and Northeast regions. Being of responsibility of cities, primary health care always underwent adversities regarding restricted availability of municipal public resources and of the low managerial and technical preparation observed in smaller cities or less economically developed regions. Incentives from the Brazilian Ministry of Health, such as Requalifica UBS (2011) and QUALIFAR-SUS (2012), have enabled the availability of resources to service restructuring. Similar to the results here found, the Northeast region has the highest number of eligible cities to receive resources from QUALIFAR-SUS^18^, a recent initiative that is still under deployment in the Country.

By analyzing *per capita* costs with medicines, Viera & Zucchi^26^ found an inverse association between expenses and city inhabitants. Cities with populations of up to 5,000 inhabitants presented a spending average *per capita* 3.9 times higher than those with 500,000 inhabitants. For the authors, the most plausible hypothesis to explain such a difference is on the purchase power of cities. Hence, one can infer that there is a smaller availability of resources destined to service structuring, as proposed by the Ordinance no. 1.555/2013^17^. Thus, the need for expanding the financing for pharmacy structuring is reinforced.

In addition to financial difficulties, the lack of equipment for service provision (chairs, service counters, computers, internet access, and computerized system) suggests that managers and professionals have difficulty in understanding pharmacies as a space destined to health service as well as medicine dispensing services as part of the health care process. The lack of these equipment prevents or restricts the development of services when it comes to its technical aspect: not having conditions of meeting patients in a individual and comfortable way or the presence physical barriers hinders the effective communication and therapeutic relationship. Communication can be even more impaired when dealing with older people, pregnant women, and patients with special needs.

Castro, Correa, and Climan^8^ point out that the place destined for meeting patients in pharmacies must have adequate reception conditions to respect the individual’s physical condition. Factors such as visual pollution, noise, and inadequate lighting might contribute to attention neglect and hinder the care process. The physical area must not represent a communication barrier.

Not having internet access and information systems isolates pharmaceutical services compared to other health services, hindering the development of the therapeutic project, as well as consultations to literature, guidelines, and medical records that support the performed services. The service must be properly structured to contribute to the solutions of the care process, as pointed out by a study^20^ on the use of computerized system and adoption of protocols for antimicrobial dispensing in the primary health care of the city of Salto Grande, São Paulo, which resulted in the decrease in the prescription and irrational use of these medicines.

The structure of health services is directly associated with receptive conditions. For Brehmer & Verdi^5^, inadequate space generates conflicting ethical situations between professionals and patients, where patients’ right to privacy and their particular needs are not addressed, thus constituting barriers in the health care service. Nora & Junges^19^, when revising the literature, found that problems concerning the environment interfere in the working process, compromising the quality of the offered services, and generating demotivation in professionals and discomfort to patients. In most of the sampled dispensing and health units, the environment does not seem to contribute to humanization as a guiding axis on the care and management practices at SUS^16^.

Campese et al.^6^ highlight that pharmacies, because of the number of establishments and the presence of pharmacists as technical manager, can be a privileged scenario when building a socially useful service aimed at health care availability.

When meeting patients, medicine delivery must no prevail as a technical and bureaucratic act. Social and technical developments of pharmaceutical services comprise effectiveness when it comes to technical results, but also to the capacity of welcoming, listening, understanding, taking responsibility, and addressing the patients’ complaints and needs. They require a humanized practice of care that not only depends on the people involved in the service (despite this being the most important component), but that also demands space and some other minimum conditions.

The lack of equipment and material resources in health units generates unfavorable working conditions^5^. Meeting patients behind a bar, standing for hours in a row, in an inconvenient environment, does not contribute to professionals’ commitment and motivation, resulting in the worsening of services. Bars or even glass, sometimes seen as way of protection for professionals from eventual conflicts with users, are a limiting factor in humanized practice of care.

The organization of pharmacies with strategically positioned barriers to ensure a certain distance between professionals and patients sometimes aims at ensuring productivity (of public and private services) and profitability (of private establishments)^6^.

The information here presented must be considered in future investments for reform and construction of new health units, as well as to define the infrastructure financing policies of services. To ensure a full and efficient health care by pharmaceutical services, it is necessary to have a pharmacy with adequate physical structure^23^.

Possible study limitations might include the loss of around 13.7% of samples of the drawn units, without compromising the validity of the sample.

Finally, our results indicate the need for environment restructuring of pharmaceutical services, aiming at humanizing care and improving work conditions for professionals. Communication barriers are frequent in the patient care of pharmaceutical services.

The reorganization of pharmacies must provide individuality and privacy, favoring the service and the direct relationship between pharmacists and patients. It also requires changes in the conceptions of medicine, from an understanding more focused on its delivery to one that sees it as part of the patient care process.

Data in the Northeast region indicate less favorable conditions in the development of adequate dispensing services, since they show smaller physical spaces, lack of equipment, and no internet access. This panorama indicates the need for incentives in the infrastructure of pharmaceutical services, considering regional specificities.

Recent regulations and incentives to the structuring of dispensing units still did not allow the total restructuring of municipal units of primary health care, indicating the need to expand the existing resources for the structuring of pharmaceutical services of Brazilian cities.

## Footnotes

a Ministério da Saúde. CONASS. CONASEMS. Nota técnica conjunta sobre qualificação da assistência farmacêutica. Brasília (DF); 2008. 3 p.
